# Decorin inhibits the migration and invasion of the LPS + high glucose–induced primary trophoblast cell through ADAMTS12

**DOI:** 10.1007/s11626-026-01166-y

**Published:** 2026-03-24

**Authors:** Qiuling Chen, Taoyan Hu, Hui Luo

**Affiliations:** Hunan Provincial Key Laboratory of Regional Hereditary Birth Defects Prevention and Control, Changsha Hospital for Maternal & Child Health Care Affiliated to Hunan Normal University, Changsha, China

**Keywords:** GDM with obesity, DCN, ADAMTS12, Primary trophoblast cells, Migration and invasion

## Abstract

Decorin was identified as the most relevant biomarker in type 2 diabetes or obesity-related diseases, but its function in gestational obesity or gestational diabetes mellitus (GDM) remains unknown. RT-qPCR or western blot were employed to validate the expression of Decorin and ADAMTS12 in the umbilical cord blood or placental tissues of the aforementioned groups. Subsequently, an LPS + high glucose–induced primary trophoblast cell model was established to verify the expression of DCN and ADAMTS12 in trophoblasts. Based on this model, silencing (si) of Decorin, or Decorin recombinant protein (DCN-r) combined with si-ADAMTS12 intervention, was performed to investigate its effects on trophoblast cell proliferation, migration, invasion, and other characteristics. Compared to the normal pregnancy group, the levels of DCN and ADAMTS12 showed an increasing trend in the gestational obesity group and the GDM group. Notably, DCN and ADAMTS12 were markedly higher in the GDM with obesity group compared to either the gestational obesity or GDM-alone groups. LPS + high glucose inhibited the migration and invasion of primary trophoblast cells while promoting increased levels of DCN and ADAMTS12. Under LPS + high-glucose intervention, DCN-r further suppressed trophoblast cell migration and invasion while enhancing ADAMTS12 expression, whereas si-DCN reversed these effects. Additionally, si-ADAMTS12 attenuated the LPS + high glucose–induced decline in trophoblast cell migration and invasion and blocked the promoting effects of DCN recombinant protein. Silencing ADAMTS12 can inhibit the promotive effect of DCN on the migration and invasion capabilities of LPS + high glucose–induced primary trophoblast cells. Clinical trial number: not applicable.

**Supplementary Information:**

The online version contains supplementary material available at 10.1007/s11626-026-01166-y.

## Introduction

Evidence from human cohort studies, epidemiological research, and animal studies indicates that maternal obesity and gestational diabetes mellitus (GDM) predispose offspring to cardiometabolic diseases (Joëlle and Wayne 2019). Furthermore, maternal obesity predisposes offspring to long-term health issues, potentially perpetuating an intergenerational cycle of obesity and insulin resistance (Paredes *et al*. 2021). Fat is no longer considered a simple storage depot but rather a dynamic organ that locally and systemically regulates energy homeostasis, glucose sensitivity, insulin resistance, and inflammatory pathways Lingling, (Kyoko *et al*. 2015). The leucine-rich small proteoglycan family protein Decorin (DCN) may play a role in the development of obesity and type 2 diabetes (T2D) by promoting adipose tissue expansion (Bolton *et al*. 2012). Decorin is highly expressed in adipose tissue and is a secreted protein associated with obesity, type 2 diabetes, and diabetic nephropathy Yasunori, (Mitsugi *et al*. 2017). In mice, the absence of decorin leads to impaired glucose tolerance, linked to increased feed efficiency and altered gene expression in adipose tissue (Svärd *et al.* 2019). These studies confirm DCN’s involvement in obesity and diabetes development, but its role in GDM patients with obesity remains unknown.

A disintegrin-like and metalloproteinase domain with thrombospondin type 1 repeats (ADAMTS) is a secreted zinc endopeptidase comprising 19 zinc endopeptidases (Li *et al.* 2022). In GDM patients, alterations in the expression, structure, and function of proteoglycans and ADAMTS proteases located in the extracellular matrix of fetal membranes (placenta, umbilical cord, amnion) lead to changes in the extracellular matrix structure (Ozler and Demircan 2017). ADAMTS7−/− ADAMTS12−/− mice exhibit abnormal collagen fibrils in tendons, accompanied by reduced levels of leucine-rich small proteoglycans (decorin, biglycan, fibromodulin) that regulate collagen fibril formation (Mead *et al*. 2018). A family-based genome-wide association study (GWAS) on childhood stroke suggested that two variants in the ADAMTS2 gene (rs469568 and rs1364044) from the ADAMTS (a disintegrin and metalloproteinase with thrombospondin motifs) gene family may play a role in childhood stroke, though their functional mechanisms remain unknown (Witten *et al*. 2020). The association of ADAMTS proteins with dysregulated coagulation signaling indicates that postnatal vascular injury and subsequent thrombosis are primary causes of childhood stroke (Arning *et al*. 2012). These studies confirm that ADAMTS12 is involved in the development of GDM and is associated with changes in DCN levels, but the roles of both in GDM patients with obesity require further investigation.

There is established evidence from in vitro trophoblast invasion models using immortalized trophoblasts that high glucose suppresses invasiveness (Belkacemi *et al*. 2005). In addition, HTR-8/SVneo cells treated with high glucose caused the accumulation of ROS and mitochondrial changes, promoting apoptosis and inhibiting migration (Sarina, Li *et al*. 2019). Our previous study has shown that LPS and 25 mM glucose induced the increase of N6-methyl-adenosine (m6A) levels in human primary villous trophoblasts (Chen *et al*. 2023). These directly supported the pathophysiological premise of the LPS + 25 mM glucose model in human primary villous trophoblasts.

Previous studies have confirmed that ADAMTS12 plays an important role in various biological and pathological processes, such as development, angiogenesis, inflammation, cancer, arthritis, and atherosclerosis (Wei *et al*. 2014). ADAMTS12 mediates communication between specific cell types and plays a critical role in regulating normal tissue development, remodeling, and degradation (Bespalova *et al*. 2012). Currently, the global prevalence of overweight and obesity during pregnancy is rising, which elevates the risk of obesity and diabetes in offspring (David and Simmons 2011; Langley-Evans *et al.* 2022). DCN can improve the function of insulin-secreting β-cells while reducing the expression of ECM proteins associated with fibrotic capsule formation (Urbanczyk *et al*. 2023). In placental hyperplasia spectrum disorders, maternal ADAMTS12 levels are elevated and positively correlate with fetal ADAMTS12 serum levels (*r* = 0.53, *p* = 0.002) (Icen Taskin 2023). Compared to both the control group and first-onset endometrial polyps (EPs) group, ADAMTS12 concentrations were significantly higher in the EPs and recurrent EPs groups (*p* < 0.05) (Nian *et al*. 2024). In contrast to early-onset preeclampsia, ADAMTS12 and sENG were significantly lower in gestational hypertension (Soobryan *et al*. 2022). These findings collectively suggest that DCN/ADAMTS12 may serve as crucial biomarkers for both the pathogenesis of GDM with obesity and early health in offspring.

Based on these findings, we collected umbilical cord blood samples from subjects with gestational obesity, GDM, and GDM with obesity for DCN and ADAMTS12 levels. Additionally, primary trophoblast cells were isolated, and an in vitro model was induced using LPS + HG. Interventions with DCN recombinant protein or ADAMTS12 gene editing were performed to elucidate the mechanistic role of DCN/ADAMTS12 in GDM with obesity. This study aims to provide reliable biomarkers and a theoretical foundation for the management and monitoring of GDM combined with obesity.

## Materials and methods

### Clinical study

According to the diagnostic criteria of the International Association of Diabetes and Pregnancy Research Groups (IADPSG), whole blood samples from normal pregnancies, patients with GDM, gestational obesity, and GDM with obesity were collected. All subjects (aged 24–30) attended Changsha Maternity and Child Health Hospital from January 2022 to January 2025 according to the IADPSG. During the experiment, all subjects informed consent to the conduct of this experiment. Clinical umbilical cord blood and placental samples from the normal pregnancies, GDM, gestational obesity, and GDM with obesity patients were collected for subsequent testing.

### Isolation, culture, and treatment of primary trophoblast cells from placental tissue

Under sterile conditions, chorionic villus tissue between the decidua layer and amniotic membrane was carefully excised. PBS was added to remove blood clots from the tissue surface. The tissue fragments were meticulously dissected using ophthalmic scissors and digested with 6 mL of trypsin (0.25%, AWC0232, Abiowell, Changsha, China). Digestion was terminated by adding 5 mL of complete medium. The cell suspension was filtered through a 40-μm strainer, followed by centrifugation at 1000 rpm for 5 min to collect the cell pellet. The cells were resuspended in D/F12 medium containing 10% fetal bovine serum + 1% penicillin and streptomycin, and cultured in T25 flasks (DH-160I, SANTN, Shanghai, China) in a 37°C, 5% CO_2_, and saturated humidity incubator. After passaging, third-generation cell climbing slices were identified by immunofluorescence (CK7) for subsequent experiments. For LPS treatment, 10 mg of the LPS (L2630, Sigma, St. Louis, MO) was added to 2 mL of H_2_O and dissolved to obtain the mother liquor with a concentration of 5 mg/mL. When in use, just dilute it by the corresponding multiple. For high glucose (HG) treatment, 20 mg of glucose was added to 4.440 mL of culture medium and dissolved to obtain the working solution with a concentration of 25 mM. Cells were treated with 200 ng/mL LPS + 25 mM HG induction + co-transfection for 48 h. Decorin recombinant protein (DCN-r) was administered at a concentration of 5 µg/mL for 48 h of co-treatment (Adam *et al*. 2012).

For transfection, the si-negative control (si-NC), si-DCN (Sense: GACUUUAUCUGUCCAAGAA/dT//dT/, Antisense: UUCUUGGACAGAUAAAGUC/dT//dT/), and si-ADAMTS12 (Sense: GGGUUGUUCCAUAACCCAA/dT//dT/, Antisense: UUGGGUUAUGGAACAACCC/dT//dT/) were provided by Sangon Biotech. Cells (1 × 10^5^ cells/well) were transfected with 3 µg of si-DCN or si-ADAMTS12 through 5 µL of Lipofectamine 2000 (11668019, Invitrogen, Carlsbad, CA).

### RT-qPCR

Total RNA was extracted from tissues using Trizol (15596026, Thermo, Waltham, MA). cDNA was synthesized using an mRNA reverse transcription kit (CW2569, Cowin Biotech, Beijing, China). The expression of target genes was detected using the UltraSYBR Mixture kit (CW2601, Cowin Biotech) and a real-time PCR system (PIKOREAL96, Thermo). The 2^−ΔΔCt^ method was employed to calculate the relative expression of target genes (Table 1).

**Table 1.** Gene sequence

Genes	Sequence	Length (bp)
DCN	F: CCTTTCCACACCTGCAAACT	199 bp
	R: GCCTCTCTGTTGAAACGGTC	
ADAMTS12	F: GTGGTGGCCGACACAAAGAT	113 bp
	R: TTGCCAATGCTTGGGTTATGG	
GAPDH	F: ACAGCCTCAAGATCATCAGC	104 bp
	R: GGTCATGAGTCCTTCCACGAT	

### Western blot

The cells or tissues were lysed with radioimmunoprecipitation assay (RIPA) buffer (AWB0136, Abiowell), and protein concentrations were determined using the BCA method. A total of 200-μg protein samples were separated by 12% sodium dodecyl sulfate-polyacrylamide gel electrophoresis (SDS-PAGE). The separated proteins were transferred onto polyvinylidene fluoride (PVDF) membranes activated with methanol and blocked with 5% skim milk (AWB0004, Abiowell), then dried at room temperature for at least 1 h. Subsequently, the membranes were incubated overnight at 4°C with primary antibodies anti-DCN (14667-1-AP, Proteintech, Chicago, IL), anti-ADAMTS12 (24934-1-AP, Proteintech, USA), and β-actin (66009-1-Ig, Proteintech) followed by incubation with secondary antibodies HRP goat anti-mouse IgG (1:5000, SA00001-1, Proteintech) and HRP goat anti-rabbit IgG (1:6000, SA00001-2, Proteintech) at 37°C for 90 min. Finally, protein bands were visualized and analyzed using ECL Plus ultrasensitive luminescence solution (AWB0005, Abiowell). The exposed photos were analyzed using quantity one professional gray-scale analysis software. The gray value of the target protein is divided by that of the internal reference protein for normalization processing to calculate the relative expression level.

### CCK8

The cells were seeded in a 96-well plate at a density of 5 × 10^3^ cells/well (100 μL per well). Then, 10 μL/well of CCK8 solution (prepared with complete medium) was added to each well, followed by incubation at 37°C with 5% CO_2_ for 4 h. Finally, the absorbance was measured using a microplate reader (MB-530, Huisong, Shenzhen, China).

### Transwell

To detect the migration ability of cells, 500 μL of complete medium containing 10% fetal bovine serum was placed in the lower chamber. Cells were adjusted to a density of 2 × 10^6^ cells/mL, and 100 μL of the cell suspension was added to each well. The plate was placed in a 37°C incubator for 48 h. The upper chamber was removed and transferred to a new well containing PBS. The upper chamber was washed three times with PBS. Non-migrated cells on the upper surface of the chamber were gently wiped off using a cotton swab. Cells were fixed with 4% paraformaldehyde (HY-Y0333, MCE, Monmouth Junction, NJ) for 20 min. The membrane was carefully detached from the chamber. The membrane was stained with 0.1% crystal violet for 5 min. The membrane was placed on a glass slide. Migrated cells on the outer surface of the upper chamber were observed and counted under an inverted microscope (DSZ2000X, Beijing Zhongxian Hengye Instruments, Beijing, China).

To detect the invasive ability of cells, 100 μL of ice-cold, serum-free DMEM-diluted Matrigel (200 μg) was added to each well and incubated at 37°C for 30 min. Subsequently, the transwell chamber (3428, Corning, NY) was used to repeat the above experimental steps for the analysis of cell invasive ability.

### ELISA

According to the manufacturer’s instructions, the level of DCN in the umbilical cord blood serum of subjects was detected using the DCN (CSB-E16522h, Wuhan Huamei Bioengineering Co., Ltd, Wuhan, China) kit.

### N6-methyl-adenosine (m6A) assay

According to the manufacturer’s instructions, changes in intracellular m6A levels were detected using the m6A (ab185912, Abcam, Cambridge, UK) assay kit.

### Data statistics

The statistical analysis in this study was performed using GraphPad Prism 8.0 software. Measurement data are expressed as mean ± standard deviation. Normality and homogeneity of variance were tested first. For data conforming to normal distribution with equal variances, an unpaired *t*-test was used for intergroup comparisons, while one-way ANOVA or repeated measures ANOVA was employed for multigroup comparisons, followed by Tukey’s post hoc test. A *p*-value < 0.05 was considered statistically significant.

## Results

### DCN and ADAMTS12 were highly expressed in the umbilical cord blood and placental tissues of GDM with obesity

**Figure 1.**
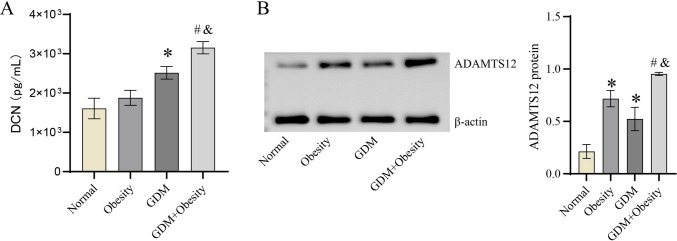
DCN and ADAMTS12 are highly expressed in the umbilical cord blood and placental tissues of GDM with obesity. (*A*) ELISA detection of DCN levels in the serum of subjects’ umbilical cord blood. (*B*) Western blot analysis of ADAMTS12 expression in the placental tissues of subjects. *p < 0.05 vs Normal, #*p* < 0.05 vs Obesity, &*p* < 0.05 vs GDM. *N* = 6 biology repeats/group. *Bar chart* with mean ± standard deviation.

Compared with the normal pregnancy group, the level of DCN in umbilical cord blood showed an increasing trend in the gestational obesity group and was significantly elevated in the GDM group (Fig. 1*A*). In comparison with the gestational obesity or GDM group, DCN was significantly higher in the GDM with obesity group (Fig. 1*A*). Further examination of placental tissues revealed that, compared with the normal pregnancy group, the protein level of ADAMTS12 was significantly increased in both the gestational obesity and GDM groups, with even higher expression of ADAMTS12 protein in the GDM with obesity group (Fig. 1*B*). These results confirm that DCN and ADAMTS12 are highly expressed in the umbilical cord blood and placental tissues of GDM with obesity.

### LPS and high glucose induce the expression of DCN and ADAMTS12 in primary trophoblast cells

**Figure 2.**
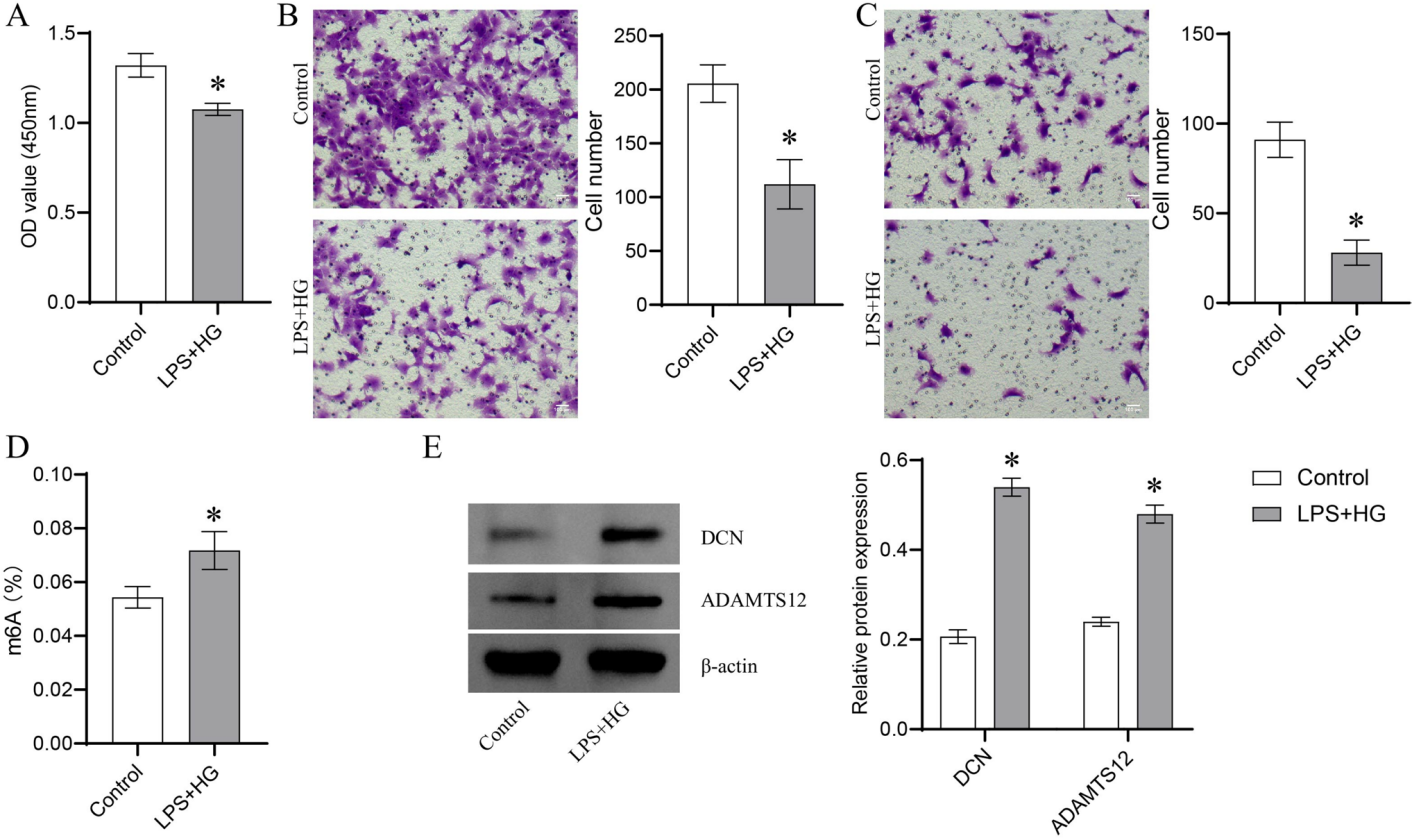
The elevated expression of DCN and ADAMTS12 in LPS + HG–induced primary trophoblast cells. (*A*) Cell proliferation was measured by CCK-8 assay. (*B*, *C*) Cell migration (*B*) and invasion (*C*) were assessed by Transwell assay. (*D*) Cellular methylation levels were detected using an m6A assay kit. (*E*) Protein expression of DCN and ADAMTS12 was analyzed by Western blot. *p < 0.05 vs Normal. *N* = 3 biology repeats/group. *Bar chart* with mean ± standard deviation.

To investigate the roles of DCN and ADAMTS12 in trophoblast cells of subjects with gestational obesity complicated by diabetes, trophoblast cells were induced with 200 ng/mL LPS + 25 mM HG for in vitro analysis. Compared with the Normal group, LPS + HG induction reduced the viability of primary trophoblast cells (Fig. 2*A*). Transwell assays showed that LPS + HG impaired the migration and invasion abilities of primary trophoblast cells (Fig. 2*B*, *C*). Additionally, LPS + HG increased the m6A levels in primary trophoblast cells compared to the Normal group (Fig. 2*D*). Furthermore, LPS + HG upregulated the protein levels of DCN and ADAMTS12 in primary trophoblast cells (Fig. 2*E*). These results confirm that DCN and ADAMTS12 expression is elevated in LPS + HG–induced primary trophoblast cells.

### Recombinant DCN protein (DCN-r) regulates the expression of ADAMTS12 in primary trophoblast cells

**Figure 3.**
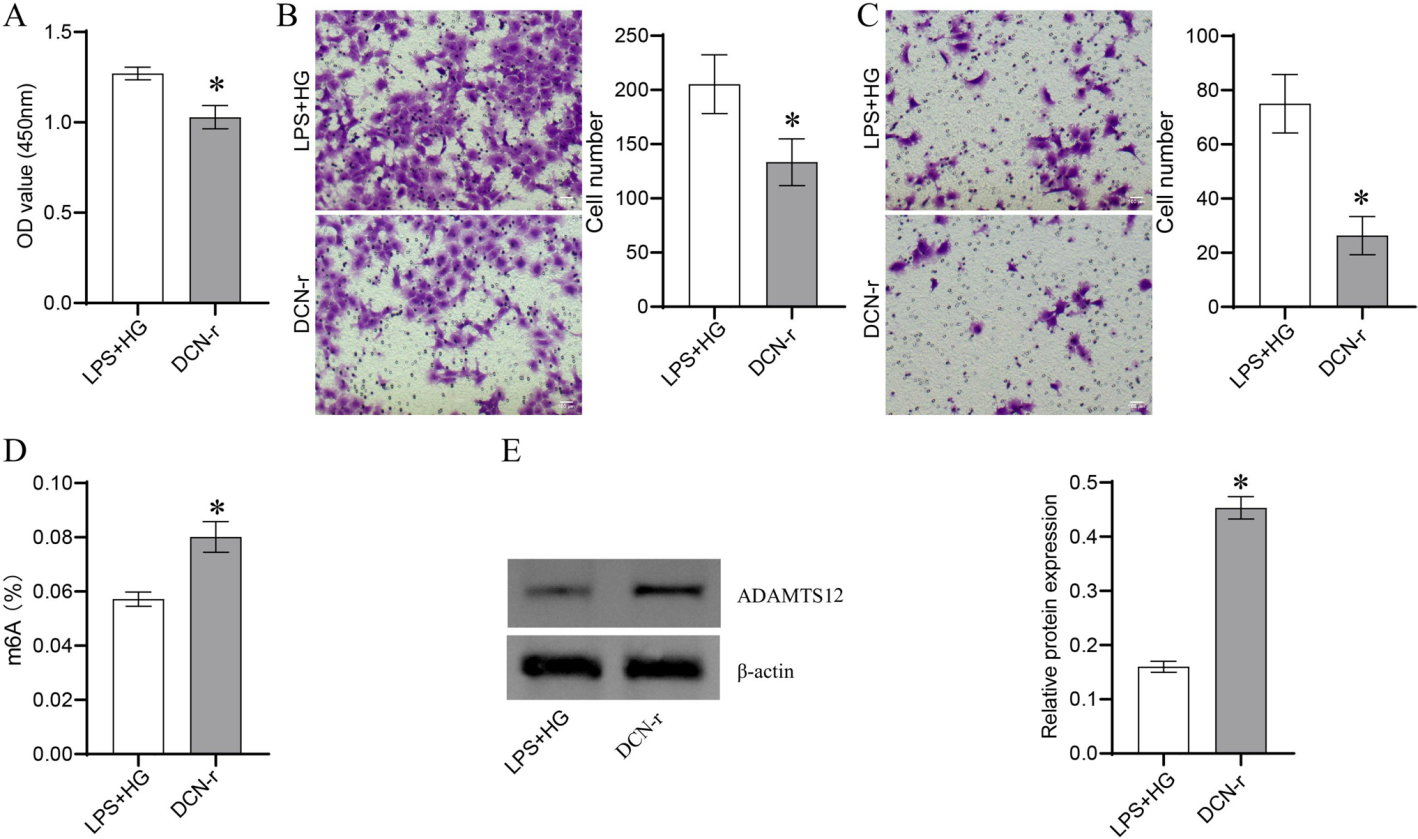
DCN-r promotes LPS + HG–induced primary trophoblast cell injury and upregulates ADAMTS12 protein expression. (*A*) Cell proliferation was measured by CCK-8 assay. (*B*, *C*) Cell migration (*B*) and invasion (*C*) were assessed by Transwell assay. (*D*) Methylation levels were detected using an m6A assay kit. *E* ADAMTS12 expression was analyzed by Western blot. *p < 0.05 vs NC. *N* = 3 biology repeats/group. *Bar chart* with mean ± standard deviation.

To investigate the roles of DCN and ADAMTS12 in LPS + HG–induced primary trophoblast cells, we treated the LPS + HG–induced primary trophoblast cells with 5 µg/mL DCN-r. The results showed that DCN-r exacerbated the reduction in cell viability induced by LPS + HG (Fig. 3*A*). Moreover, DCN-r treatment further inhibited the migratory and invasive capacities of primary trophoblast cells under LPS + HG stimulation (Fig. 3*B*, *C*). DCN-r reduced the m6A levels in LPS + HG–induced primary trophoblast cells (Fig. 3*D*). Further analysis revealed that DCN-r upregulated ADAMTS12 protein expression in LPS + HG–induced primary trophoblast cells (Fig. 3*E*). These findings demonstrate that DCN-r aggravates LPS + HG–induced damage in primary trophoblast cells and upregulates ADAMTS12 protein expression.

### Silencing DCN regulates the expression of ADAMTS12 in primary trophoblast cells

**Figure 4.**
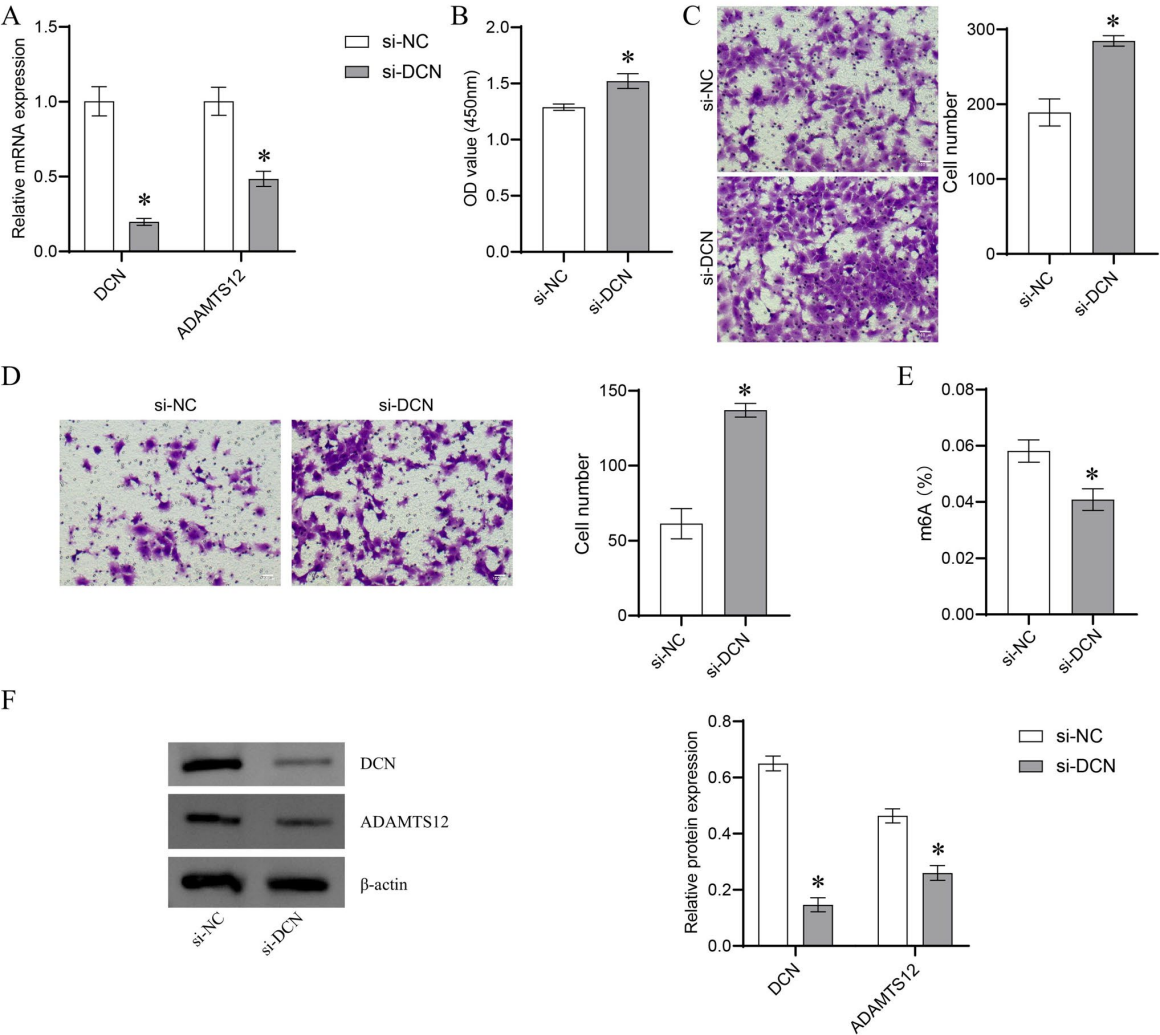
Silencing DCN alleviates LPS + HG–induced damage in primary trophoblast cells and downregulates ADAMTS12 protein expression. (*A*) RT-qPCR analysis of DCN and ADAMTS12 expression. (*B*) CCK-8 assay for detecting cell proliferation. (*C*, *D*) Transwell assay for assessing cell migration (*C*) and invasion (*D*). (*E*) m6A assay kit for measuring cellular methylation levels. (*F*) Western blot analysis of DCN and ADAMTS12 expression. *p < 0.05 vs si-NC. *N* = 3 biology repeats/group. si-NC refers to LPS + HG–treated cells transfected with a negative-control siRNA. *Bar chart* with mean ± standard deviation.

Primary trophoblast cells were further transfected with silenced DCN and simultaneously treated with LPS + HG for 48 h. The results showed that compared with the si-NC group, si-DCN inhibited the mRNA expression of DCN and ADAMTS12 in primary trophoblast cells (Fig. 4*A*). Further detection revealed that DCN silencing promoted the increase in cell viability of LPS + HG–induced primary trophoblast cells (Fig. 4*B*). Transwell assays demonstrated that DCN silencing facilitated the recovery of migration and invasion capabilities in LPS + HG–induced primary trophoblast cells (Fig. 4*C*, *D*). Additional tests indicated that DCN silencing upregulated the m6A methylation levels in LPS + HG–induced primary trophoblast cells (Fig. 4*E*). In LPS + HG–induced primary trophoblast cells, transfection with si-DCN downregulated DCN and ADAMTS12 protein expression (Fig. 4*F*). These results demonstrate that DCN silencing inhibits LPS + HG–induced damage in primary trophoblast cells while downregulating ADAMTS12 protein expression.

### DCN regulates ADAMTS12 to aggravate LPS + HG–induced primary trophoblast cell injury

**Figure 5.**
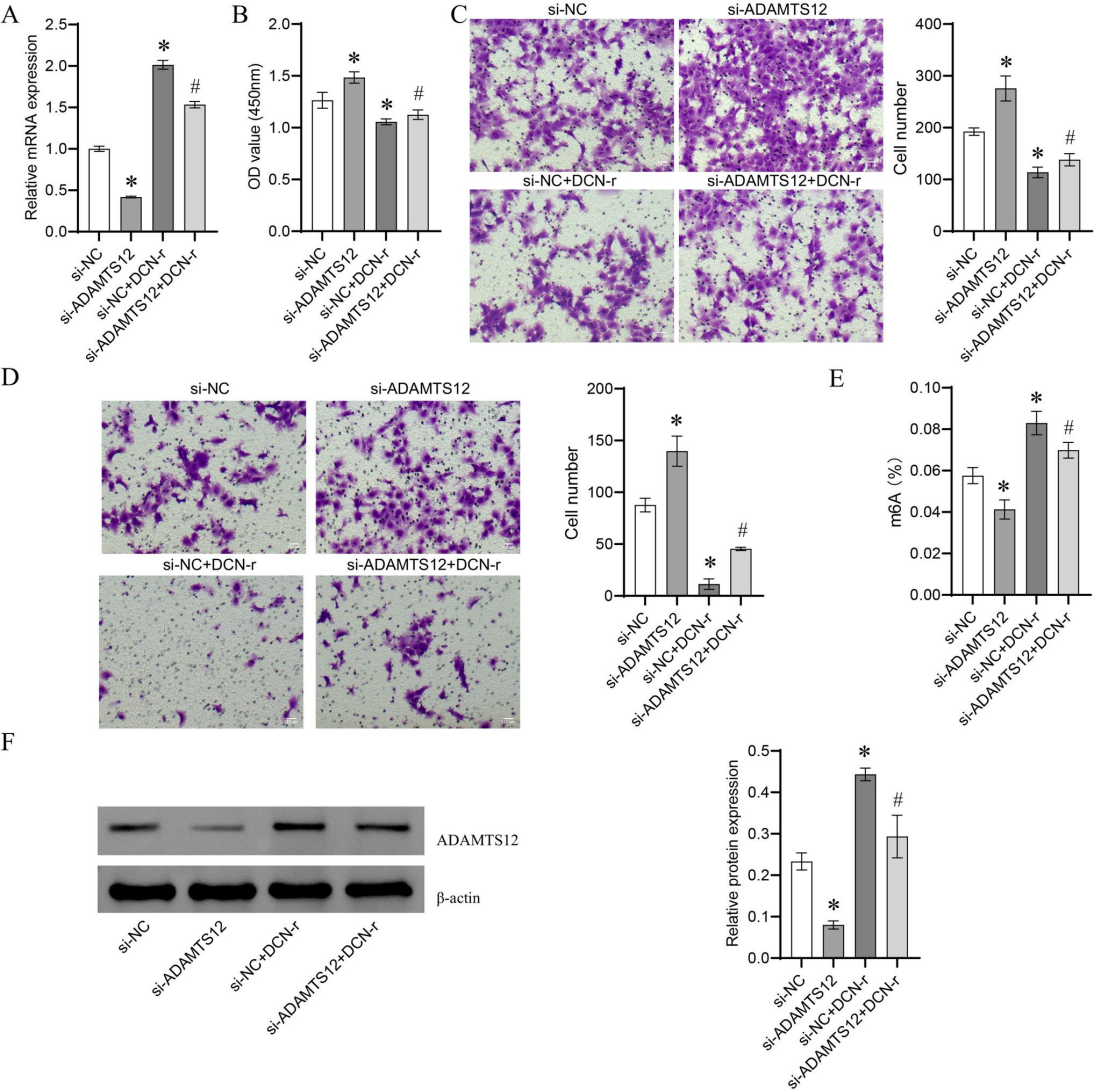
Silencing ADAMTS12 blocks the promoting effect of DCN on LPS + HG–induced damage in primary trophoblast cells. (*A*) ADAMTS12 expression detected by RT-qPCR. (*B*) Cell proliferation detected by CCK-8 assay. (*C*, *D*) Cell migration (*C*) and invasion (*D*) detected by Transwell assay. (*E*) Cellular methylation level detected by m6A assay kit. (*F*) ADAMTS12 expression detected by Western blot. *p < 0.05 vs si-NC, #*p* < 0.05 vs si-NC + DCN-r. *N* = 3 biology repeats/group. si-NC refers to LPS + HG–treated cells transfected with a negative-control siRNA. *Bar chart* with mean ± standard deviation.

Compared with the si-NC group, ADAMTS12 silencing inhibited the expression of the ADAMTS12 gene in LPS + HG–induced primary trophoblast cells, while DCN-r promoted its expression (Fig. 5*A*). When DCN-r was administered to LPS + HG–induced primary trophoblast cells, concurrent si-ADAMTS12 intervention blocked the promotive effect of DCN-r on ADAMTS12 gene expression (Fig. 5*A*). ADAMTS12 silencing attenuated the LPS + HG–induced decline in viability of primary trophoblast cells (Fig. 5*B*). When DCN-r was administered to LPS + HG–induced primary trophoblast cells, concurrent si-ADAMTS12 intervention abolished the exacerbating effect of DCN-r on cellular injury (Fig. 5*B*). Transwell assays demonstrated that ADAMTS12 silencing promoted the migration and invasion of LPS + HG–induced primary trophoblast cells (Fig. 5*C*, *D*). When DCN-r was administered to LPS + HG–induced primary trophoblast cells, concurrent si-ADAMTS12 intervention promoted the migration and invasion of trophoblast cells (Fig. 5*C*, *D*). ADAMTS12 silencing downregulated the m6A levels in LPS + HG–induced primary trophoblast cells (Fig. 5*E*). When DCN-r was administered to LPS + HG–induced primary trophoblast cells, concurrent si-ADAMTS12 intervention downregulated m6A levels (Fig. 5*E*). Compared with the si-NC group, ADAMTS12 silencing suppressed the expression of ADAMTS12 protein in LPS + HG–induced primary trophoblast cells, whereas DCN-r enhanced its expression (Fig. 5*F*). When DCN-r was administered to LPS + HG–induced primary trophoblast cells, concurrent si-ADAMTS12 intervention counteracted the stimulatory effect of DCN-r on ADAMTS12 protein expression (Fig. 5*F*). These results confirm that ADAMTS12 silencing can block the promotive effects of DCN on the migratory and invasive capacities of LPS + high glucose–induced primary trophoblast cells.

## Discussion

Our clinical-level findings demonstrated that compared to the normal pregnancy group, umbilical cord blood DCN levels showed an increasing trend in the gestational obesity group and were significantly elevated in the GDM group. Notably, DCN levels were markedly higher in the GDM with obesity group compared to either the gestational obesity or GDM-alone groups. Western blot analysis of placental tissue indicated that ADAMTS12 protein levels were significantly increased in both the gestational obesity and GDM groups relative to normal pregnancies, with even higher expression observed in the GDM with obesity group. The m6A methylation METTL3/IGF2BP2 axis promotes osteoarthritis progression by upregulating ADAMTS12 expression and enhancing STAT1 stability and expression (Yang *et al*. 2023). Our preliminary studies confirmed elevated methylation levels of the ADAMTS12 in placental tissue from obese pregnant women with diabetes. Therefore, further investigation into m6A methylation modifications of ADAMTS12 may provide novel insights into its role in GDM with obesity.

The onset and progression of diabetic osteoarthritis and osteoarthritis are closely associated with the synthesis and metabolism of extracellular matrix components (such as fibronectin and decorin) (Zhao *et al*. 2022). Our preliminary studies confirmed that compared to the control group, the concentration of the endogenous metabolite hesperidin was upregulated in the umbilical cord blood of the obese group (Chen *et al*. 2022). Endogenous hesperidin forms hydrogen bonds and hydrophobic interactions with the ATG7 protein and inhibits the m6A levels in human primary villous trophoblasts induced by LPS and 25 mM glucose (Chen *et al*. 2023). Furthermore, in vitro experiments demonstrated that LPS + high glucose suppresses the migration and invasion of primary trophoblasts, promotes increased levels of DCN and ADAMTS12, and elevates m6A levels. These findings suggest that endogenous metabolism in patients with gestational diabetes mellitus and obesity may potentially correlate with DCN/ADAMTS12 expression, warranting further investigation in subsequent studies.

It has been reported that the expression of Decorin is significantly upregulated in the adipose tissue of obese mice with impaired glucose tolerance and type 2 diabetes, primarily localized in the stromal/vascular cells of adipose tissue (Bolton *et al*. 2008). Decorin gene therapy alleviates diabetic cardiomyopathy and improves left ventricular function, which may be associated with reduced cardiac inflammation and fibrosis (Chen *et al*. 2020). In the non-obese diabetic (NOD) mouse model of Sjögren’s syndrome, proteolytic degradation of decorin is elevated in exocrine tissues and correlated with increased TGF-β levels in gland lysates and saliva (Yamachika *et al*. 2000). Our results demonstrate that, under LPS and high-glucose intervention, DCN-r further suppresses trophoblast cell migration and invasion while promoting ADAMTS12 expression, whereas si-DCN reverses this effect. These findings suggest that DCN drives trophoblast cell proliferation by regulating ADAMTS12 expression, thereby mediating the progression of GDM complicated by obesity.

Functionally, both DCN and ADAMTS12 are upregulated to participate in extracellular matrix formation (Ho *et al*. 2017). DCN may inhibit high glucose–induced apoptosis and oxidative stress damage in lens epithelial cells by partially suppressing the p22phox-p38 pathway (Du *et al*. 2020). Overexpression of DCN can improve diabetic cardiomyopathy and promote angiogenesis through the IGF1R-AKT-VEGF signaling pathway Jinsheng, (Fuqiong *et al*. 2017). The reduced endothelial wall integrity conferred by ADAMTS variants, along with inflammatory processes and defective vascular remodeling, plays a significant role in the pathogenesis of cerebral aneurysms (Arning *et al*. 2016). Our study demonstrates that si-ADAMTS12 suppresses the decline in migration and invasion capabilities of trophoblast cells induced by LPS + high glucose, and also prevents the harmful effects of DCN-r. These results indicate that silencing ADAMTS12 counteracts the driving role of DCN in GDM complicated by obesity, providing a new theoretical foundation for the management and treatment of this disease.

However, due to the limitation of experimental funds, this study did not further analyze the specific molecular mechanism by which DCN regulates the expression of ADAMTS12, which is the limitation of this research. DCN-r increased ADAMTS12 expression, and si-DCN reduced it, indicating that DCN acts upstream of ADAMTS12. In addition, si-ADAMTS12 weakened the inhibitory effects of DCN-r on trophoblast migration and invasion, showing that ADAMTS12 mediates the function of DCN under LPS + high-glucose conditions. Given the changes in m6A levels during DCN intervention, we hypothesize that DCN may bind to M6A-modified proteins to mediate the expression of ADAMTS12. On the other hand, it remains unclear whether DCN, as a secreted protein, is negatively regulated by ADAMTS12. This may require further RNA or proteomic sequencing to analyze the further interaction and regulation between the two. This is the key direction for analysis in the subsequent research and also our next work plan.

In summary, our study confirms silencing ADAMTS12 blocks the promotive effect of DCN on the migration and invasion capabilities and m6A levels of LPS + high glucose–induced primary trophoblast cells. This research provides a new theoretical foundation for the management of GDM with obesity.

## Supplementary Information

Below is the link to the electronic supplementary material.

ESM 1 (DOCX 1.64 MB)

## Data Availability

The data used to support the findings of this study are available from the corresponding author upon request.
