# Supplementary figures and images for: Decorin inhibits the migration and invasion of the LPS + high glucose–induced primary trophoblast cell through ADAMTS12
Source: In Vitro Cell Dev Biol Anim. 2026 Mar 24;62(5):639–48. doi: 10.1007/s11626-026-01166-y (PMC13246882; doi:10.1007/s11626-026-01166-y)

Figure 1B


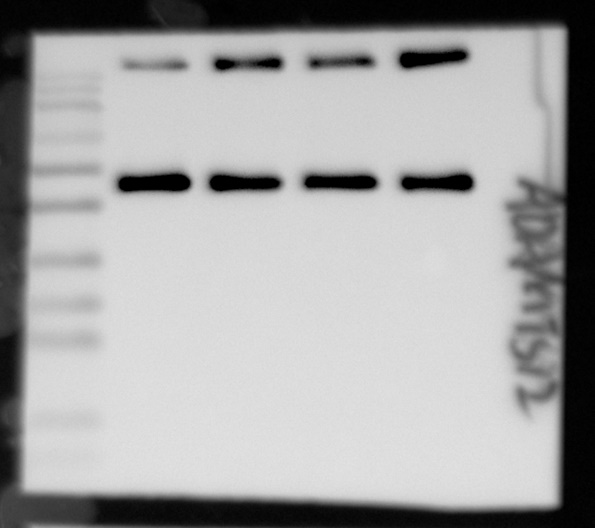


Figure 2E


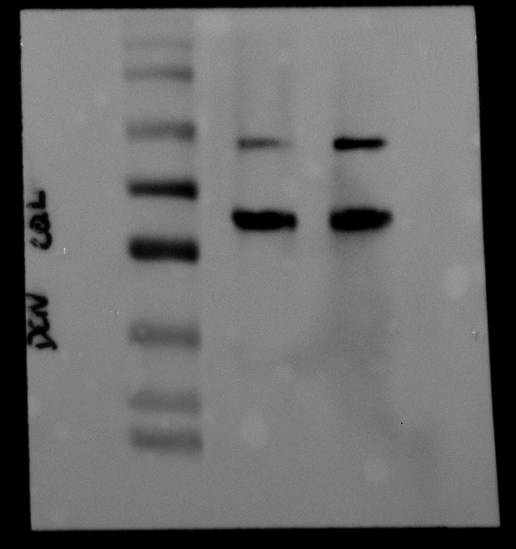


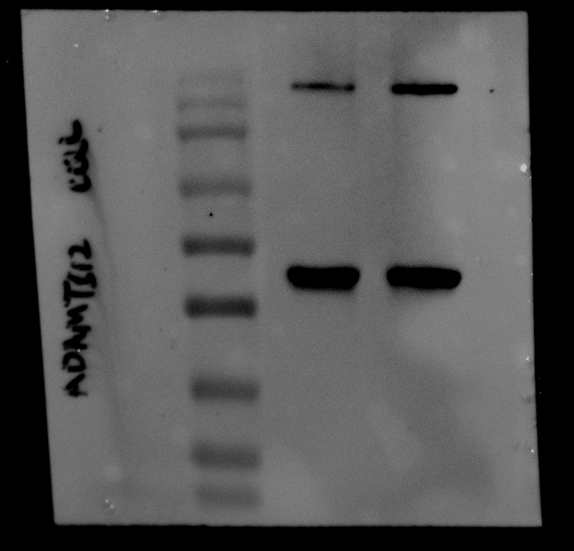


Figure 3E


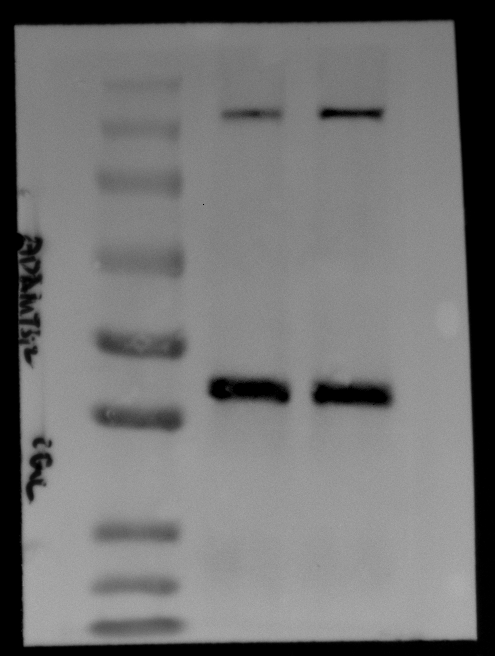


Figure 4F


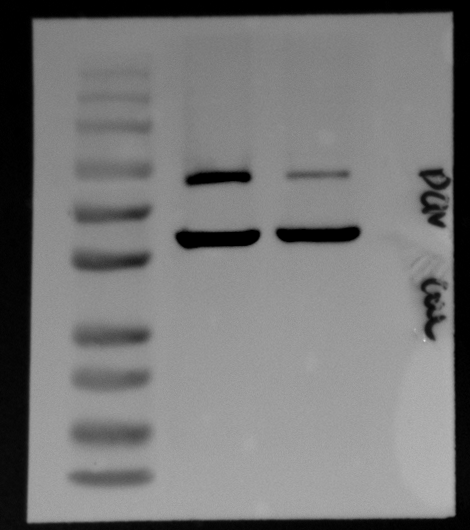


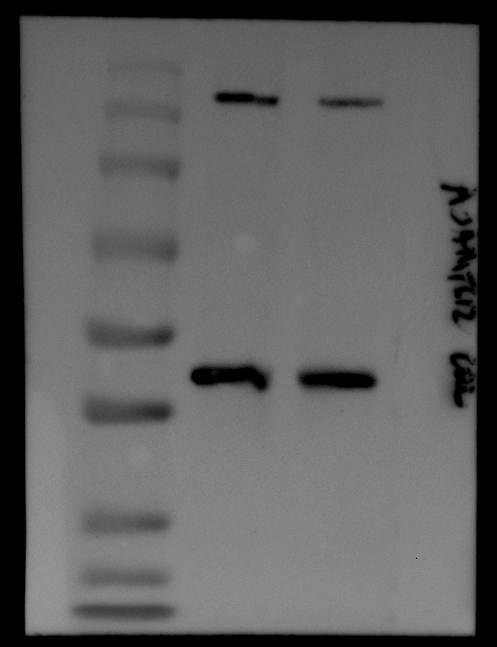


Figure 5F


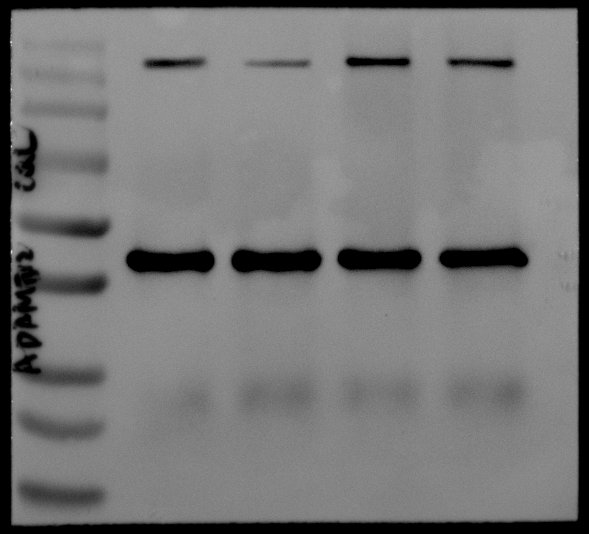

Supplement: Supplementary file 1 — (DOCX 1.64 MB) [file 11626_2026_1166_MOESM1_ESM.docx]
